# The Role of Natural Antioxidants Against Reactive Oxygen Species Produced by Cadmium Toxicity: A Review

**DOI:** 10.34172/apb.2020.023

**Published:** 2020-02-18

**Authors:** Velid Unsal, Tahir Dalkıran, Mustafa Çiçek, Engin Kölükçü

**Affiliations:** ^1^Faculty of Health Sciences and Central Research Laboratory, Mardin Artuklu University, Mardin, Turkey.; ^2^Department of Pediatric Intensive Care, Necip Fazıl City Hospital, 46030, Kahramanmaras, Turkey.; ^3^Department of Anatomy, Faculty of Medicine, Kahramanmaraş Sütçü imam University, Kahramanmaras, Turkey.; ^4^Department of Urology, Faculty of Medicine, Gaziosmanpasa University,Tokat, Turkey.

**Keywords:** Cadmium, Oxidative stress, ROS, Natural antioxidants

## Abstract

Cadmium (Cd) is a significant ecotoxic heavy metal that adversely affects all biological processes of humans, animals and plants. Exposure to acute and chronic Cd damages many organs in humans and animals (e.g. lung, liver, brain, kidney, and testes). In humans, the Cd concentration at birth is zero, but because the biological half-life is long (about 30 years in humans), the concentration increases with age. The industrial developments of the last century have significantly increased the use of this metal. Especially in developing countries, this consumption is higher. Oxidative stress is the imbalance between antioxidants and oxidants. Cd increases reactive oxygen species (ROS) production and causes oxidative stress. Excess cellular levels of ROS cause damage to proteins, nucleic acids, lipids, membranes and organelles. This damage has been associated with various diseases. These include cancer, hypertension, ischemia/perfusion, cardiovascular diseases, chronic obstructive pulmonary disease, diabetes, insulin resistance, acute respiratory distress syndrome, idiopathic pulmonary fibrosis, asthma, skin diseases, chronic kidney disease, eye diseases, neurodegenerative diseases (amyotrophic lateral sclerosis, Parkinson’s disease, Alzheimer’s disease, and Huntington disease). Natural antioxidants are popular drugs that are used by the majority of people and have few side effects. Natural antioxidants play an important role in reducing free radicals caused by Cd toxicity. Our goal in this review is to establish the relationship between Cd and oxidative stress and to discuss the role of natural antioxidants in reducing Cd toxicity.

## Introduction

### Cadmium

Cadmium (Cd) is a bright silvery-white soft metal in group II B of the periodic table. Cd has an atomic number of 48, an atomic weight of 112.41 u and a density of 8.64 g/cm^3^.^1,2^ Cd is not pure in nature. However, it was purified for the first time in 1817 and had a commercial designation in the early 1900s.^3^ Present in the ground at a rate of 0.15-0.2 mg/kg, Cd is obtained as a by-product during the decomposition of zinc ore.^4^ In the United Kingdom and the United States, some black clay deposits contain high levels of Cd, and there is Cd at high concentrations in the ground. The main source of Cd atmospheric release is volcanic activity. It is thought that the amount of Cd released by volcanic activity is between 100-500 tons. Volcanic movements in deep seas are also one of the natural sources of Cd.^5^

### Cd usage areas

Cd is an easily formable metal due to its physically soft nature. It has a wide range of applications since it has excellent properties against corrosion and has important properties in metal usage such as low melting temperature.^6^ Cd is most commonly used in the construction of nickel-Cd batteries.^7,8^ Cd is widely used as a coating material in PVC and shipbuilding industry due to its resistance to oxidation. Cd sulfur compounds are also used as color materials in the production of plastic, glass, ceramic, rubber, paint and fireworks.^9,10^ In addition, household goods, automobiles and trucks, agricultural tools, aircraft parts, industrial tools, hand tools and fasteners (screw nuts, bolts, screws and nails) are generally covered with Cd. It is also used in tire repair and photography.^11^

### The effects of Cd

In humans, Cd exposure is due to food, inhalation and predominantly cigarette smoke.^12^ Exposure through the skin is less. Cadmium chloride (CaCl_2_) is the major form of water-soluble Cd in high concentrations and is highly well absorbed from the mouth. Cadmium oxide (CaO) is the most common form exposed through respiration.^13^ Many occupational groups are exposed to significant proportions. Among these occupational groups, workers in mining, paint and battery factories are particularly exposed to significant amounts of Cd dust and fumes. Following these groups, smokers are those most exposed to Cd.^14^ One cigarette contains 1.5-2 μg of Cd. About 10% of it goes to cigarette smoke, and during smoking, 50% of this cigarette is absorbed by the lungs. Approximately 1 μg of Cd enters the body of a person smoking 20 cigarettes a day.^15^ Studies have shown that the Cd levels of smokers are 3-4 times higher than those of non-smokers.^16,17^ In non-smokers, Cd exposure is usually linked to food intake. The daily amount of Cd consumed by foodstuffs is about 10-25 μg but may vary with environmental Cd rates. For example, in Japan, the amount of Cd taken by food is 28 μg/day compared to 9.9 μg/day in China and 9-10 μg/day in Germany.^18^ In contaminated areas, Cd intake with food can reach several hundred micrograms per day. Drinking water usually contains Cd in low quantities, and the approximate amount is 1 μg/L or less.^19,20^ Cd is one of the most effective carcinogenic metals for humans. Cd and its compounds have been described among human carcinogens in 1993 by the International Organization for Cancer Research.^21^ Cd affects cellular proliferation, differentiation, apoptosis and other cellular activities.^22^ Prolonged exposure to Cd can result in lung, prostate, testicular, and kidney cancer. Because the half-life of Cd is 10 to 30 years, the effect of this metal on it may be carcinogenic.^23^ Inactivation of tumor suppressor genes causes the deterioration of cell adhesion mechanism, triggering of apoptosis, suppression of DNA repair, free radical production and affecting the antioxidant system.^24,25^ Cd can easily penetrate the body and pass into the cytoplasm via the calcium channels through the cell membrane. It can then bind to intracellular molecules, accumulate in the cell, cause metabolic transformation, or be dislodged. High levels of Cd are bound by Cd by glutathione, sulfhydryl-rich soluble proteins and metallothioneins (MTs) in the cell to try to reduce the intracellular and extracellular fluid levels.^26^ Cd causes the release of free oxygen radicals such as superoxide (O2-), hydroxyl (OH-), nitric oxide (NO) and hydrogen peroxide (H_2_O_2_) in the organism like other heavy metals.^27^

Cd causes peroxidation in membrane lipids, degradation of the antioxidant defense system, the emergence of inflammation, protein structure disorders and the oxidation of nucleic acids, and it negatively affects the DNA repair mechanism. Cd is a very potent toxic metal that indirectly contributes to the production of free oxygen radical species.^28-30^

### Cd and reproductive system

Exposure to Cd causes a decrease in reproductive performance evaluates such as fertility, abnormal embryonic development, prenatal death and sexual dysfunction.^31^ Prolonged exposure may cause structural and functional disturbances in the male and female reproductive system.^32,33^ In males, it causes a decrease in sperm motility and spermatogenesis index.^34^ Acute Cd-induced damage to the testes manifests itself with hemorrhagic inflammation, organ degeneration and dysfunction and vacuolization of the seminiferous tubules. It has been reported that after high doses of Cd, the seminiferous tubule diameter and the tubular volume density decrease markedly.^32^ In one study, 39.98% of the reproductive capacities of rats that received Cd for 52 weeks orally were found to be reduced.^35^ In experimental studies on female rats, it has been reported that Cd ovulation is inhibited and that it directly reduces progesterone production by acting directly on granulosa cell morphology and steroid biosynthesis.^36,37^ Another in vivo study on female rabbits reported that when Cd chloride was administered at a dose of 1.5 mg/kg, there was a decrease in the number of primary follicles and an increase in the number of atretic follicles in the ovary.^38^

### Cd and cardiovascular system

Cd can be stored in the heart, like kidney, liver. However, when compared to the kidney and liver, it has been reported that the concentration of Cd in the heart tissue is relatively low. In a study in rats, 50 ppm Cd^+2^ per day was applied to rats’ diets for 7 weeks. Studies have reported that Cd^+2^ accumulation occurs in cardiac tissues at ratios of 0.55-1.22 μg.^39^ Cd has effects on cardiac tissue. Two important hypotheses have been proposed regarding this topic: the effects of Cd on cardiac tissue structure and integrity and its effects on the cardiac conduction system.^40^ The first is based on increased reactive oxygen species (ROS) with increased oxidative stress and necrosis and ultrastructural changes in cells with increased ROS. The second is based on the interaction of Cd with contraction and acceleration systems.^41,42^ Cd has been shown to have the potential to serve as a new risk factor on the cardiovascular system, as demonstrated by in vitro animal studies and human studies.^43^ Studies show that Cd leads to metabolic and structural disorders in the heart and that it plays a role in the etiology of hypertension even at low concentrations.^44-46^ In a recent study, Cd content in serum, hair, and nails of patients with hypertensive and coronary heart disease was compared to those of a healthy control group, and as a result, it was reported that Cd content increased in various tissues of the patient group.^47^ Another study found that urinary Cd, the biological indicator of long-term Cd exposure, was associated with increased cardiovascular mortality and increased incidence of cardiovascular disease.^48^ These studies support that Cd exposure is a cardiovascular risk factor. However, there are also publications that show the opposite. Vivoli et al reported that urine and hair Cd contents in the hypertensive patient group were similar to those of normotensive individuals, but urinary Cd/copper ratio was significantly lower in hypertensive patients.^49^ Cd causes atherosclerosis. It has been reported that the incidence of atherosclerosis is increased in people living in areas with Cd contamination. These findings may shed light on the etiology of atherosclerosis, which is common in smokers.^50^

There are various hypotheses on this subject. According to the information available, the dominant hypothesis explaining the effect of Cd on atherosclerosis is the “response to injury hypothesis” proposed by Ross. According to this hypothesis, it is a functional disorder that initiates primary endothelial damage and/or diseases. Cd reduces endothelial barrier function by causing degradation of endothelial cell-cell adhesions, and death of endothelial cells was demonstrated in vivo and in vitro.^51^

### Cd and placenta

The placenta is a versatile organ that protects the fetus and plays an important role in its development and in placenta functions between mother and fetus during pregnancy. Some lesions developing on the placenta may adversely affect fetal life.^52-54^ In addition, it acts as a filter in reducing the passage of harmful substances and protects the embryo and then the fetus without exposure to contaminants.^55^ Cd can pass through the mother and to the fetus through the placenta.^56-57^ Human placenta is sensitive to toxic Cd activity. Experimental studies have shown the administration of Cd salts in the late period of pregnancy to cause placental damage. Early exposure to Cd in particular is thought to have an impact on baby health, such as neurological, developmental and endocrine disorders.^55^ Cd also affects endocrine hormone synthesis (eg, placental progesterone or leptin) and changes trophoblast cell migration.^58^ In one study, there was a significant negative correlation between the concentration of Cd in the cord blood and TSH concentration in the neonatal blood.^59^ In addition, Cd can damage the fetus by affecting the metabolism of elements such as zinc, copper, iron and selenium.^14^

### Cd and brain

Cd enters the central nervous system either by smell or by altering the permeability of the blood-brain barrier. Cd causes neurotoxicity with complex pathology-involving behavioral changes, brain biochemical defects, and neurological dysfunction.^60^ In the central nervous system, Cd causes oxidative stress and histologically observable membrane disorders due to the decrease of acetylcholinesterase activity, the increase of oxidative stress symptoms and the depletion of glutathione, superoxide dismutase and other antioxidants.^61^

### Cd and kidney

The kidney is one of the major organs affected by different ways of exposure to Cd. Whether acute or chronic, it is adversely affected in all circumstances. In chronic Cd exposure, approximately 50% of the accumulated dose is stored in the kidneys.^62,63^ Acute toxicity occurs when too much Cd is inhaled or taken orally. Acute exposure rarely results in death. The exposure resulting in death is 20-30 mg/kg for humans.^64,65^ Exposure to Cd-oxide vapors at doses of 5 mg/m^3^ for 8 hours has been reported to be lethal to humans.^66^ This stored amount represents the amount of Cd not bound to MT. The S-1 segment of the kidney proximal tubules is the main target site for Cd deposition. Cd inhibits reabsorption of protein, amino acid, glucose, bicarbonate and phosphate in the proximal tubules, resulting in tissue damage. It induces apoptosis in tubular cells, causing oxidative stresses in transport proteins and mitochondria.^67,68^ Cd disrupts vitamin D metabolism in the kidneys, causing a devastating effect on the bones. This effect causes absorption of calcium from the intestines and impairs collagen metabolism, thus resulting in osteomalacia and osteoporosis. The most important example of this is the *Ittai Itai* disease in Japan. Renal tubular dysfunction, impaired calcium absorption, anemia and osteomalacia cause severe pain in this disease.^69,70^ The earliest sign of kidney damage is the asset of low molecular weight proteins. These are B2 microglobulin (b2M), retinol binding protein (RBP) and enzymes like N-acetyl-β-D-glucosaminidase (NAG). Detection of b2M and RBP in urine provides information about impairment of proximal tubular cell function. Thus, the urinary RBP and b2M are defined as biological markers of proximal tubular dysfunction.^64,71^

### Cd and endocrine system

Cd has adverse effects on the endocrine system. According to previous research, hormones are affected negatively.^72,73^ Endocrine disrupting chemicals are natural or synthetic agents that mimic, enhance or inhibit the effects of endogenous hormones, and recent reports suggest that Cd may be added to endocrine disrupting chemicals as it has the potential to mimic the estrogenic effects of various tissues.^74,75^ Jancic and Stosic reported that Cd tends to accumulate not only in the liver, kidneys, or other organs but also in the thyroid gland. They found that the concentration of Cd in the blood is positively correlated with accumulation in the thyroid gland.^76^ Chronic exposure to Cd causes many histological and metabolic changes in the thyroid gland.^77^ In a study, elevated blood levels of Cd have been associated with suppressed TSH production. However, the increase in Cd to urine ratio has also been correlated with increase in triiodothyronine (T3) and thyroxine (T4) serum levels.^78^ In a rat experiment, it was determined that Cd caused calcitonin, synaptophysin, chromogranin A and somatostatin secretion. Ca^+2^ levels in the serum decreased considerably in these animals. In addition, Cd may impair the metabolism of Ca, as well as other basic metals such as Zn, Se and iodine and the structure and function of thyroid follicular cells in female rats chronically exposed to Cd.^76,79^

### Cd and liver

The liver plays an important role in maintaining body homeostasis in living organisms. Plasma proteins have functional properties such as construction, regulation of blood composition, detoxification and hormone inactivation.^80,81^ The liver is the organ most affected by Cd through all exposure patterns. Cd-induced hepatotoxicity depends on the amount and duration of exposure. As in other tissues and organs, histopathological and metabolic changes occur in the liver, along with some histopathological changes such as loss of normal architecture of the parenchymatous tissue, cytoplasmic vacuolization, cellular degeneration and necrosis, congested blood vessels, destructed mitochondria cristae, fat globules, severe glycogen depletion, lipofuscin pigments, and collagenous fibers.^82,83^ These changes may result in both apoptosis and necrosis.^83,84^ Mitochondrial-mediated apoptosis may be involved in metal-induced cell deaths. Hepatotoxicity is thought to be caused mainly by the binding of Cd to thiol groups in the mitochondria, leading to mitochondrial dysfunction and related injury.^83^ Cd triggers a programmed creation of necrotic cell-killing by the rupture of lysosomes.^41^ Increased serum concentrations of amino acids in the urea cycle have been reported in individuals exposed to Cd via diet. This is also an indication of kidney damage.^85^

Plasma ALT, AST and GGT enzyme levels are known to be the most important indicators for evaluating the structural and functional status of liver tissue.^86^ Cd causes structural and functional impairment in cells by increasing lipid peroxidation. Cd also leads to an increase in blood enzyme levels by transferring these enzymes to the blood and disrupting the membrane permeability of the cells.^87,88^ Oxidation of lipids results in degradation of the membrane structure by degradation and crosslinking of polymerization.^89^

### Cd and bone

Recent studies have established a target for Cd in the bone even at low exposures, as in other tissues and organs.^14,90^ Chronic Cd exposure is associated with bone loss, low bone mass, and an increase in fracture incidence. The function of osteoblasts and osteoclasts, and the cells associated with the bones that resemble the bone include development, repair, and skeletal renewal, respectively. Adult skeleton is interestingly dynamic, with the rebuilding of the bone in response to mechanical and metabolic demands. It is not known what effect Cd has on bone loss at the cellular level. Under comparable incubation conditions in organ and cell culture systems, bone resorption appears to be more sensitive than bone formation versus Cd exposure. Cd toxicity reduces phosphate uptake of Fibroblast growth factor 23 (FGF-23) in bones and causes osteomalacia along with phosphaturia.^91^ FGF-23 plays an important role in balancing mineral ion homeostasis and bone mineralization.^92^ Cd is toxic to an osteoblast variety, MC3T3, by an unknown mechanism and activates osteoclasts and causes osteoporosis.^93-94^ In a study on rats, Cd has been reported to reduce serum osteocalcin levels.^95^ In children exposed to Cd ‘calciuria’, increased bone resorption and reduced bone mineral concentration have been observed.^96^ It has been reported that administration of Cd to rats at a dose of 0.5 mg/kg three times a week increased the risk of osteoporosis by reducing the biomechanical quality of the bone.^97^ In another animal study, single-dose oral Cd has been shown to increase calcium excretion in mice. Similar studies have shown that Cd destroys the collagen matrix and causes calcium to be released from bone to blood.^98^

### Cd and hematopoiesis

Cd toxicity negatively affects hematopoiesis. Anemic hemolysis due to Cd toxicity has been observed. In fact, severe anemia was observed in patients with Itai-itai due to Cd toxicity, which is linked to significant suppression of erythropoietin production.^99^ Three mechanisms have been proposed to explain why Cd causes anemia.

1. Hemolysis due to deformities of peripheral red blood cells.^100^
2. When iron is absorbed in the duodenum, iron deficiency develops due to Cd competing with iron.^101^
3. Renal anemia due to hypo-production of erythropoietin^99^ along with these, at high doses, acute Cd has been shown to increase the rate of polychromatic erythrocytes by showing a genotoxic effect in bone marrow.^101^

Acute Cd toxicity also causes pial cerebral thrombosis by accelerating platelet aggregation.^89^

### Cd and immune system

Cd exposure changes the immune system.^102^ It has been reported that Cd intoxication in the prenatal period disrupts T lymphocyte production and immunization in the postnatal period and causes abnormal thymocyte development.^103,104^ In rats receiving chronic Cd toxicity (40 mg/L/30 days), tumor necrosis factor alpha (TNF-α), interleukin-1β, 6, 10 (IL-1β, 6, 10), peripheral neutrophil levels and interferon gamma (IFN-γ) increased, while lymphocyte counts decreased.^105^ In addition to autoimmune formation, lymphocyte proliferation and natural killer cell activity are also suppressed.^106^

### Cd and lung

The lungs are among the target organs of Cd. Exposure to Cd dust or vapor by respiration irritates the respiratory tissues of humans. The most important source of inhalation Cd poisoning is cigarette smoke. Human lungs reabsorb more than almost half of the Cd (40-60%) in tobacco smoke.^107^ Male rats fed orally with CdCl_2_ for 10 days showed a decrease in lung weight due to the dose. However, the same situation was not observed in female rats. Nonspecific pulmonary lesions were seen in male rats fed with 1.2 mg/kg CdCl2 in water for 200 days.^108^ Cd may also contribute to asthma symptoms. Zn and Selenium (Se) can treat the clinical symptoms of asthma such as wheezing, coughing and lung function by competing with and removing Cd in children. Despite the limited number of studies showing that Cd has no association with lung cancer, recent studies have identified an increased risk of lung cancer in the population exposed to Cd.^109,110^ It has been reported that Cd levels in 24-hour urine are positively associated with both lung cancer and total cancer risk.

### Cd and Cancer

Cd is one of the most effective carcinogenic metals for humans. Chronic exposure to Cd causes lung, prostate, breast, pancreatic and renal cancer.^69,111,112^ The cellular and molecular mechanisms of the carcinogenic effect of Cd are given under six forms as below.^110^

1) Activation of proto-oncogenes.

2) Inactivation of tumor suppressor genes.

3) Deterioration of cell adhesion mechanism.

4) Induction of apoptosis.

5) Inhibition of DNA repair.

6) Free radical production and affecting the antioxidant system.

Cd promotes the expression of genes (such as c-myc, c-fos, and c-jun) that are members of the activator protein 1 (AP-1) family of proto-oncogenes. In addition, with immediate early response genes, MT increases the expression of stress response genes that encode glutathione and heat shock proteins, suppresses the expression of antioxidant genes such as superoxide dismutase, catalase and glutathione peroxidase. Tumor suppressor proteins such as E-cadherin and VE-cadherin impair cell-cell adhesion.^113-115^

### Cd and trace elements, metallothioneins

Since Cd has toxicological properties, it can prevent the uptake, transport and use of many other elements.^116^ Se is a trace element with antioxidant effects based on the human body that shows oxygen-free radical cleaning, protects organs and tissues in the body from oxidative damage and improves the immune system of the body. Previous in vitro studies have shown Se to be a protective agent against Cd cytotoxicity by blocking ROS production. In addition, Se has been shown to stimulate antioxidant enzymes in the immature kidneys of rats exposed to Cd and to protect against oxidative damage.^117^ Zn plays a role in many cellular functions, catalytic functions of many enzymes and structural stability of various cell proteins. Zn also interacts in DNA/RNA binding by the regulation of transcription, chromatin structure, protein-protein interactions and RNA metabolism. It also has an important role in stabilization and protection of biological membranes against oxidative and peroxidative injuries, loss of plasma membrane integrity and change of membrane permeability. Zn supplementation at low concentrations has been shown to reduce Cd-induced oxidative stress.^118^ Another study reported Zn supplementation to be beneficial for the system against Cd toxicity.^116^ Environmentally essential toxic metal Cd is present as Cd^+2^ ion in biological systems and is chemically similar to Ca^2+^ in such cases. Both are bivalent and can enter cells through channels or protein-bound penetration. Cd disrupts calcium homeostasis by inhibiting calcium channels and/or related proteins. Altered calcium homeostasis induced by Cd results in cell apoptosis, autophagy or tumorigenesis.^119,120^ Metals, hormones, cytokinins, various chemicals, inflammation, stress and hypoxia, necrosis factors and glucocorticoids induce synthesis of MTs.^121^ With their rich thiol groups, MTs are proteins involved in many physiological and pathological events, mainly antioxidant processes. They are involved in many important events, such as the detoxification of heavy metals such as Hg, Cd and lead (Pb), the regulation of essential metals such as Cu and Zn, antioxidant action against oxygen radicals and protection against DNA damage, maintenance of cell viability, angiogenesis, apoptosis and proliferation functions.^122^ MTs are thought to play a role in the homeostasis of essential metals such as Zn, Cu and Fe. MTs binds Zn, but Zn can easily be replaced with excess Cu, Fe or Cd.^123^ MTs are zinc-containing proteins consisting of 33% cysteine amino acid. MTs protect tissues from the toxic effects of heavy metals that have toxic effects in biological systems such as Cd and Hg. While MT has minimal effects on the absorption of Cd from the intestine, it plays an important role in Cd retention in tissues and reduces the excretion of Cd into bile. Excessive amounts of MT-containing cells are resistant to and are protective against Cd toxicity.^124-126^

## Oxidative stress

Oxidative stress occurs when the balance between ROS and the antioxidant system is impaired in the oxidant direction. Oxidative stress is a natural process, and there are specialized mechanisms that control this stress. Oxidative damage occurs when these mechanisms are inadequate.^127^ The cellular and molecular mechanisms of Cd can be categorized as follows: inactivation of tumor suppressor genes, deterioration of cell adhesion mechanism, triggering of apoptosis, suppression of DNA repair, free radical production and effect on antioxidant systems.^32,33^ Most of the damage caused by toxic metals is due to the increase in the free radicals they cause. ROS can lead to oxidative stress within cells by reacting with macromolecules and causing damages. These damages are in the form of increased lipid peroxidation, DNA damage, and oxidation of proteins.^83^

### ROS

ROS production plays an important role in Cd toxicity, and various suggestions have been put forward in this regard. It has been suggested that acute Cd toxicity mechanisms involve the depletion of glutathione and protein-bound sulfhydryl groups, resulting in increased production of ROS such as superoxide ion, hydrogen peroxide and hydroxyl radicals. Cd-enhanced ROS causes lipid peroxidation and results in DNA damage. Another suggestion is that mitochondria are an important target of Cd toxicity. It has been suggested that Cd initially binds to protein thiols in the mitochondrial membrane, affects mitochondrial permeability transmission, inhibits respiratory chain reaction and then produces ROS. Cancer, diabetes, atherosclerosis, neurodegenerative diseases and many other diseases play a role in the pathogenesis of ROS. For this reason, it is one of the most studied topics in recent years. ROS is a non-stable, highly effective atom or molecule having one or more unpaired electrons. Free radicals are produced according to endogenous or exogenous factors.^127-129^

### Potential endogenous sources of ROS

Mitochondria, endoplasmic reticulum, cytochrome P-450, peroxisomes, microsomes, and inflammatory cell activation are potential sources of ROS. Mitochondria are the main source of superoxide (O_2_^.–^) in the body. For each mg of protein, it produces 2-3 mol O_2_ per minute while also producing H_2_O_2_. When cytochrome P450 is induced, it specifically produces O_2_- and H_2_O_2_. Xanthine oxidase (XO), NADPH oxidase, lipoxygenase and other oxidant enzymes are important ROS sources. The ROS family includes short-lived molecules that result from the reduction of molecular oxygen. The most common ROS are H_2_O_2_, O_2_^.–^ and OH⁻.^130,131^

### Potential exogenous sources of ROS

- Antineoplastic agents: Doxorubicin, an anticarcinogenic agent, inhibits DNA replication in the cell. This leads to the formation of H_2_O_2_ and O_2_- and ultimately the initiation of lipid peroxidation.
- Radiation and environmental agents [air pollution, heavy metals, pesticides, cigarette smoke, solvents, anesthetics, aromatic hydrocarbons, Carbon tetrachloride (CCl^4^), Iron nitrilotriacetate (Fe-NTA)] cause free radical formation.^132,133^
- Habitual substances: Alcohol and narcotics.
- Environmental agents: Xenobiotics (air polluting photo chemicals, hyperoxia, pesticides, cigarette smoke, solvents, anesthetic substances, aromatic hydrocarbons).
- Stress: The level of catecholamine increases in stress. Oxidation of catecholamines is a source of free radicals.
- Ischemia, trauma.^134^

**Figure 1:**
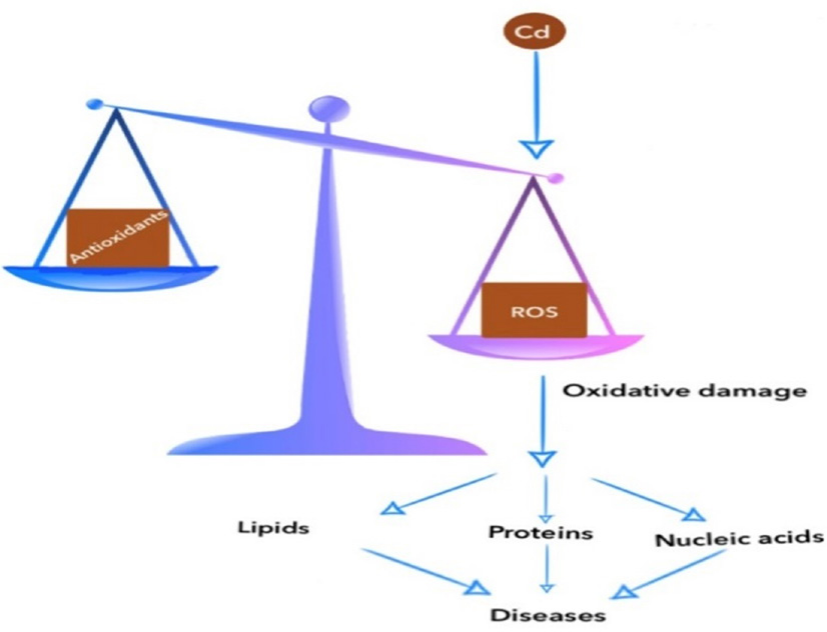
Demonstration of the relationship between ROS and antioxidants. Oxidative stress occurs when the balance between ROS and the antioxidant system is impaired in the oxidant direction. ROS affect all important structures such as carbohydrates, lipids, Nucleic acids and enzymes. Cd: Cadmium, ROS: Reactive oxygen species.

ROS attack protein and enzymes, carbohydrates, and DNA in the cell membrane lipids, causing DNA damage, as well as protein modification^131^ (Figure 1).

### Antioxidant defence system

**Figure 2:**
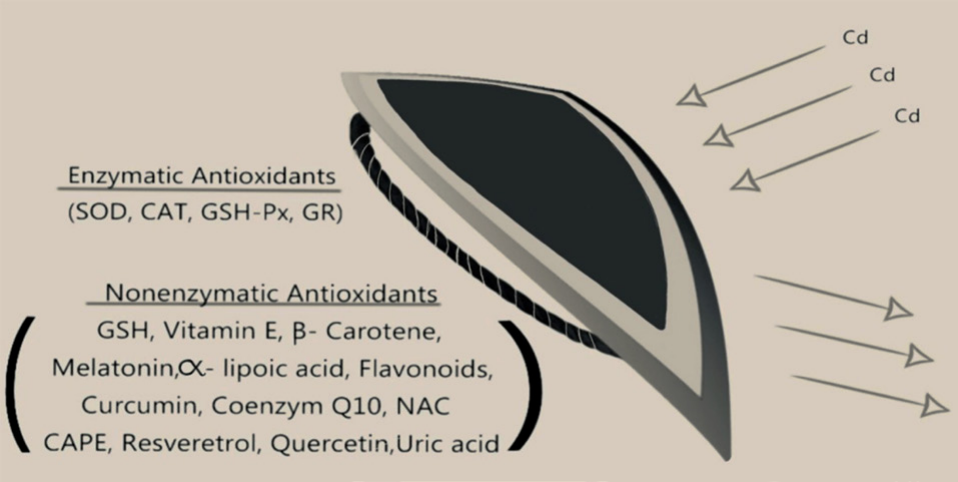
There are two types of the antioxidant defense systems. The first is the enzymatic antioxidant system, and the other is the nonenzymatic antioxidant system. Both serve as shields against the harmful effects of Cd. SOD: Superoxide dismutase, CAT: Catalase, GSH-Px: Glutathione peroxidase GR: Glutathione reductase, GSH: Glutathione, NAC: N-acetylcysteine, CAPE: Caffeic acid phenethyl ester.

Antioxidants and ROS penetrate the whole of life and constitute the field of redox biology. The antioxidant system prevents radical formation before damage, repairs the oxidative damage, cleans the damaged molecules and prevents mutations.^135,136^ Two different mechanisms play an active role against free radical damage. The first is the non-enzymatic nutritional mechanism, and the other is the enzymatic mechanism (Figure 2). In the non-enzymatic feeding mechanism, glutathione (GSH) is present in some trace elements as well as low molecular weight molecules such as vitamin E, vitamin C, beta-carotene and vitamin A. Vitamins act as the carrier or silent receptors of free radicals, whereas trace elements regulate the activities of antioxidant enzymes.^127^ Antioxidant defense systems of the human body are complex and are classified in various forms. The final classification involves their functions, their place in the cell, the structure they protect, their solubility and their source.

### Superoxide dismutase (SOD)

SOD is the most important enzyme that protects against O_2_^.–^ radicals. SOD catalyzes the conversion of superoxide to oxygen and hydrogen peroxides. The SOD enzyme family is named according to the cofactors used to detoxify the excess of O_2_^.–^ such as Cu/Zn-SOD, Fe-SOD, Ni-SOD and Mn-SOD. It is defined as metalloenzymes because it contains metals. Cu/Zn-SOD is in a dimeric form and is found in cytosol, with Cu and Zn bound to two subunits. SOD is usually found in the mitochondria and is tetrameric.^135,136^ The presence of specific SOD isoforms in different subcellular compartments emphasizes the need for tight control of ROS homeostasis.^137-139^ The role of SOD has been recognized as an important factor in oxidative stress caused by Cd. It has been shown in *in vivo* and *in vitro* studies that Cd reduces SOD activity. SOD with no activity or decreased activity causes an increase of ROS.^139^ SOD activities have been reported to decrease in the liver and kidneys of rats that were sacrificed 24 hours after a single dose of 2.5 mg/kg and 5 mg/kg Cd.^140^

### Catalase (CAT)

CAT is a potent antioxidant enzyme in protein structure. CAT is a hemoprotein with four heme groups in its structure. The molecular weight of hemoprotein CAT is 248 kDa.^141^ The most important task of CAT is to remove toxic H_2_O_2_ from cells. CAT, an enzyme found in plants, animals and aerobic bacteria, is found mostly in peroxisomes in the cell. A molecule CAT can convert 6 million hydrogen peroxide molecules per minute into water and oxygen. It has high activity in erythrocytes, kidney and liver. In vivo and in vitro studies have shown that CAT activity changes in the negative direction due to interaction with Cd.^142^

### Glutathione peroxidase (GSH-Px)

The GSH-Px enzyme is key in the living organism antioxidant system under both normal and oxidative stress conditions. GSH-Px has great important defenses against free oxygen radicals, peroxides and carcinogens. GSH-Px also plays an important role in inhibiting tumor formation by altering the lipoxygenase and cyclooxygenase pathways. In tumors, enzyme defense against H_2_O_2_ and other peroxides have been reported to cause significant increase in GSH-Px activity, the first enzyme.^131^ The GSH-Px enzyme, which catalyzes the reduction of peroxides, converts reduced GSH to oxidized glutathione (GSSG) in the cell. It has been reported that there are 8 GSH-Px in the human organism, and it is called selenoproteins (Se GPxs), which are seen in many different cell types. Only GSH-Px-4, GSH-Px-7 and GSH-Px-8 are monomeric, while the others are tetrameric. GSH-Px can be found in two forms: selenium-bound and not selenium-bound. The selenium-bound group consists of four members that reduce hydrogen peroxide and other organic peroxides. These are GSH-Px1, GSH-Px2, GSH-Px3 and GSHPx4.^143^ GSH-Px1 or cellular GSH-Px (cGSH-Px) is a cytosolic enzyme in the tetrameric structure found in all cells. GSH-Px1 is active against organic hydroperoxides and H_2_O_2_. This enzyme has been found in all tissues studied, albeit much less in germ cells.^144^ GSH-Px 2 or gastrointestinal GSH-Px (GSH-Px-GI) is found in the liver and gastrointestinal tract in humans, but not found in the kidney, heart and lung.^145^

GSH-Px 3 or plasma GSH-Px (pGSH-Px) are found in the kidneys and are especially present in the epithelial cells of proximal tubules.^143^ GSH-Px4 or phospholipid GSH-Px (PH-GSH-Px) is found in cytosol, mitochondria and cell membrane. The enzyme reduces the phospholipid hydroperoxides to alcohols and protects the membrane against peroxidation in the absence of the most important antioxidant vitamin E.^146^ Cd decreases GSH-Px activity in induced cells. The reduced activity of GSH-Px can be explained by the sulfur competition of Cd-metallothionines and GSH-Px containing amino acids.^147,148^

### Glutathione reductase (GR)

GR is responsible for providing reduced glutathione. It is one of the most abundant thiols in most cells. In its reduced form, glutathione plays an important role in the cellular control of ROS.^146^ It is involved in the re-conversion of GSSG into reduced GSH by the reducing power of NADPH.^148^

### Glutathione S-transferase (GST)

GST is a multifunctional enzyme that provides homeostasis in the detoxification metabolic pathway by catalyzing the first step in the formation of the water-soluble end product mercapturic acid. GST enzymes are a multifunctional polymorphic isoenzyme family that protect the cell from cytotoxic and genotoxic stresses.^149^ The most common tissues of the GST are the liver, especially organs such as kidney, small intestine, intestine, lung and breast. The basic biological roles of GSTs include detoxification and protection against oxidative stress. GSTs have four families: cytosolic, mitochondrial, microsomal/membrane-associated and fosfomycin/glyoxalase. There are 17 different cytosolic GSTs divided into 7 classes (alpha, mI, omega, phi, sigma, theta and zeta) based on sequence similarity in humans.^150^

### Non-enzymatic antioxidants

- Those in the lipid phase (α-tocopherol, β-carotene)
- Those found in a liquid phase (cell cytosol or blood plasma) (ascorbic acid, urate, cysteine, ceruloplasmin, transferrin, lactoferrin, myoglobin, hemoglobin, ferritin, albumin, bilirubin, glutathione)
- Those present in both liquid and lipid phase (melatonin).

### Glutathione

GSH is a tripeptide with a nucleophilic structure and strong antioxidant properties. The glutathione system acts as the major redox buffer in most cells. Decreased GSH in tissues can lead to peroxidative tissue damage with the deterioration of the cellular defense mechanism against ROS. In addition, glutathione has a key role in protecting against xenobiotics and heavy metals such as Cd.^151-153^

### Vitamin E

Vitamin E is a vitamin type that has a high level of antioxidant potency and is soluble in oil. Vitamin E has eight isoforms, α-, β-, γ-, and δ-tocopherol and α-, β-, γ-, and δ-tocotrienol stereoisomers including α, β, γ, δ tocopherol and α, β, γ, δ tocotrienol. Among these stereoisomers, the most biologically active is α-tocopherol.^154,155^ The most important property of alpha-tocopherol is its ability to protect against lipid peroxidation and to protect the cell membrane from degenerative effects of free radicals. Vitamin E has been reported to be protective against colon and prostate cancer, some cardiovascular diseases, ischemia, cataract, arthritis and neurological diseases as a result of the studies done.^154^ In vivo studies have shown that Vitamin E is protective against Cd toxicity and reduces oxidative damage.^155,156^

### β–Carotene

Carotene is a chemical terpene with two main types: alpha-carotene (α-carotene) and beta-carotene (β-carotene). There are also gamma, delta and epsilon- (γ, δ and ε-) carotenes.

β-carotene is most commonly available in green fruits and vegetables. β-carotene is particularly abundant in carrots.157 Being a good antioxidant, it has an important effect in eliminating harmful free radicals. In many studies, β-carotene has been shown to reduce oxidative stress damage and to play a protective role against oxidative stress.^157,158^ Studies have shown β-carotene to reduce oxidative stress caused by Cd-induced brain and testicular toxicity and to improve the loss of cellular antioxidants in these tissues.^159,160^

### Melatonin

Melatonin is a neurohormone that is synthesized and secreted by the retina, bone marrow, thymus, ovary and gastrointestinal tract, primarily the pineal gland.^161^ In the skin, it is responsible for the protection of deep tissues against the harmful radiation effects of sun rays and the exchange of pigment granules. On the other hand, it plays a role in the protection of oxidative cholesterol derivatives and bile acid to oxidative damage to the bile and mucous membranes.^162^ Since melatonin can be dissolved in both liquid and lipid phases, it can easily reach all intracellular components and effectively protect the cell membrane, organelles and nucleus from free radical damage. It also reduces the production of radicals such as O_2_, H_2_O_2_ and OH⁻ that occur in mitochondrial respiration. Its ability to reach the core ensures that DNA is protected against oxidative damage.^163^ There are in vitro studies suggesting that melatonin has protective effects on Cd-induced cancer cell proliferation. Furthermore, in vivo studies have shown that melatonin reduces the toxic effects of Cd.^84,164^

### α-Lipoic acid (ALA)

ALA is a small molecule that contains a thiol group and has antioxidant activity. It is efficient in both lipophilic and hydrophilic environments. Although ALA is found in human diet in sufficient quantities, mitochondria are synthesized de novo by lipoic acid synthase. ALA is found in two forms as oxides and dihydrolipoic acid as in its reduced form. Although both forms are active, it is stated that the most active form is dihydrolipoic acid. However, both forms are antioxidant. In addition to the antioxidant effects that ALA possesses, it strengthens and renews the intracellular levels of other antioxidants (vitamins C, E, GSH, etc). Because an antioxidant neutralizes its free radical, it loses its antioxidant property. Studies have shown that treatment with ALA leads to modulatory effects in Cd toxicity and reduces Cd destructive effects, leading to marked reductions in Cd residues in the liver and kidneys. In conclusion, it is claimed that ALA may be used as a potential therapeutic agent against Cd-induced toxicity.^165-167^

### Flavonoids

Flavonoids are a large group of polyphenolic compounds found in plant foods consumed regularly (i.e. vegetables and fruits), olive oil and drinks such as tea and wine. Flavonoids are known as phenylpropanoids with important chelating and antioxidant properties and are generally found in plants and cannot be synthesized in the human organism. Flavonoids are classified as flavones (grapes, French bean seeds, rice bran, vetch), flavonols (quercetin, rutin, strawberries, apples, persimmons, onions, cucumbers), flavanones (lemons, rosehips, bitter orange, petit grain, orange, orange juice) flavanonols (vinegar), flavanols or catechins (tea leaves, black tea, oolong tea), anthocyanins (cranberries, blueberries, plums, grapes, cherries, sweet potatoes) and chalcones. It has been found that flavonoids have different properties such as antiviral, antiallergic, antitumor, anticholinesterase, antithrombotic, anti-inflammatory and vasodilating effects besides antioxidant properties. Flavonoids provide protection against damage caused by Cd with these effects.^168,169^ The cytoprotective effects of flavonoids against Cd-induced diseases have been explained through specific mechanisms. First, they clean the ROS of flavonoids, reducing lipid peroxide production and increasing the activity of antioxidation enzymes. Second, they chelate Cd, reducing Cd deposition and altering in vivo levels of other basic metal ions. Third, they reduce DNA damage and prevent apoptosis.^170^

### Curcumin

Curcumin is a hydrophobic polyphenol compound used as a yellow spice for years and is obtained from turmeric. Curcumin has antioxidant, antimicrobial, anti-inflammatory, antiviral, anti-carcinogenic and antiapoptotic properties.^171^ It has been shown to be an important agent against Cd-induced hepatotoxicity, immunotoxicity, lung diseases, reproductive toxicity, neurotoxicity, colon toxicity and nephrotoxicity.^172^

#### Antioxidant effect

Curcumin exhibits potent antioxidant activity comparable to vitamins E and C. The antioxidant effect comes from its phenolic structure and β-diketone derivative. Curcumin has been shown to inhibit lipid peroxidation, H_2_O_2_ and enhance the activity of antioxidant enzymes (SOD, GST, and GPx).^173,174^

#### Anticancer effect

In vivo and in vitro studies have shown curcumin to have an inhibitory effect in three steps of carcinogenesis: tumor increase, angiogenesis and tumor growth. It has been shown to protect against tumors in many animal studies. This protection has been shown in colon, prostate, duodenum, esophagus, stomach and oral cancers.^175^

### Anti-inflammatory effect

Various studies have shown that curcumin modulates the production of a variety of inflammatory cytokines and thus exhibits a potent anti-inflammatory activity. In addition, it has an inhibitory effect on proinflammatory cytokines TNF-α and IL-6, which are inflammatory process inhibitors caused by Cd poisoning.^176^

### Coenzyme Q10 (CoQ10)

CoQ10 is a vitamin-like compound that can dissolve in oil, can exist in all cells and acts as a coenzyme during key enzymatic interaction in forming energy in the cell. The chemical formula of CoQ10 consists of 5-ethyl-2.3-dimethoxy-6-decaprenyl-1.4-benzoquinone. It is synthesized from phenylalanine and mevalonic acid endogenously in the body. The majority of CoQ10 in cell membranes is a reduced form (KoQH2) and is found near the unsaturated lipid chains in membranes. CoQ10 interacts with ROS to prevent protein and lipid peroxidation from initiating and damaging biomolecules. CoQ10 (also known as ubiquinone), an important electron carrier in cellular respiration, exhibits antioxidant properties by removing ROS and protecting oxidative stress cells. The mitochondrial respiratory chain acts as the electron. In addition, the membrane stability of CoQ10 also functions in cell signaling, gene formation, cell growth and apoptosis protection.^177-179^

In Cd poisoning study; Cd caused significant changes in hematological and biochemical parameters and increased lipid peroxidation, altered the glutathione circuit and decreased the activity of antioxidant enzymes. CoQ10 provided protection by substantially normalizing these values and reducing lipid peroxidation. The results of this study showed that CoQ10 may be useful in the prevention of Cd-induced hepatotoxicity and can be used as a preventive against acute Cd poisoning of exposed persons.^180^

### N-Acetyl cysteine (NAC)

NAC is an N-acetylated derivative of a thiol molecule L-Cysteine, a natural amino acid. Its chemical formula is C5H9NO3S, and it has a molecular weight of 163.2 g/mol. Acetylcysteine is a mucolytic that reduces the viscosity of secretions. The drug can be administered orally, intravenously or by the respiratory route. NAC is used intravenously in the treatment of paracetamol (acetaminophen) poisoning, while it is used as the second choice in acrylonitrile and methacrylonitrile poisoning. It has begun to be used in some psychiatric and physiological diseases due to the demonstration of its antioxidant properties. NAC is very effective in neutralizing free radicals in vitro. NAC reacts rapidly with hydroxyl radicals. It is also effective against other ROS such as O_2_^•–^ and H_2_O_2_. It increases SOD activity and GSH levels and inhibits autocatalytic lipid peroxidation. One study showed NAC to improve Cd-induced neurotoxicity and improve memory and learning processes of Cd-poisoned rats.^181,182^

### Caffeic acid phenethyl ester (CAPE)

CAPE is an active component with a flavonoid-like structure found in propolis produced by honey bees and has anti-inflammatory, neuroprotective, hepatoprotective, immunomodulatory, antiviral, anticancer, antioxidant and apoptosis-regulating properties.^183,184^ More than 180 substances were found in the structure of propolis. Among these components, flavonoids, caffeic acid and esters are the most dense and active constituents. CAPE can also be chemically synthesized as a result of esterification of caffeic acid and phenethyl alcohol with acid.^183-187^ CAPE consists of two ring structures. In these rings, there are two functional OH groups that carry almost all the chemical properties of the molecule, and they actively play a role in oxidation and reduction. The aromatic and aliphatic structure has a lipophilic character for carrying very long carbon groups. It is a potent inhibitor of enzymes such as ornithine carboxylase, 5-α reductase, protease, cyclooxygenase, lipoxygenase, xanthine oxidase and HIV-1 integrase. In one study, pretreatment with CAPE improved all changes caused by Cd. The results of this study showed CAPE to play a promising role as a mitochondrial-targeted antioxidant against the renal toxicity of Cd.^184,188^

### Resveratrol

Resveratrol is one of the most widely studied phytochemicals with beneficial effects on human health. In recent years, pharmacological effects of resveratrol in relation to cardiovascular health, diabetes, aging, obesity and cancer have been demonstrated by various biochemical mechanisms in preclinical models.^189^ Resveratrol has been reported to play an antiproliferative role on various types of cancer such as breast, lung, prostate, colorectal and stomach cancer, esophageal tumors, pancreatic cancer and leukemia. The anti-cancer activity of resveratrol is mediated through the modulation of various cell-signaling molecules that regulate the cell cycle process, proliferation, apoptosis, metastasis, angiogenesis and invasion of cancer cells.^190,191^ There are studies showing that resveratrol inhibits tumor growth in cancerous cells by inhibiting NF-κB activation and decreasing TNF-α production.^192^

Resveratrol has different mechanisms of antioxidant effect. Resveratrol shows an antioxidant potential by eliminating free radicals, binding metal ions, reducing the activity of enzymes involved in ROS formation and increasing antioxidant enzyme activities.^193,194^ Resveratrol inhibits lipid peroxidation caused by free radicals and prevents DNA damage. It has been reported that resveratrol lipid peroxidation is more effective when compared to vitamins E and C in prevention and that it maintains cell viability and inhibits oxidation by targeting molecules in the cell.^195^ Resveratrol has been shown to improve the renal oxidative damage caused by Cd.^196^ In addition, in another study, resveratrol has been shown to have a protective role in the amelioration of mitochondrial health through Sirt3-dependent FoxO3a.^197^

### Quercetin

Quercetin contains three rings and five hydroxyl groups in its structure. Taken at a daily dose of 50-500 mg, quercetin has many important functions for metabolism such as antioxidant, anticancerogenic, antiviral, antithrombotic, anti-ischemic, anti-inflammatory and antiallergic properties. Quercetin is known as the most powerful antioxidant substance in flavonoids. Quercetin protects the cells against endogenous and exogenous ROS tissue damage.^198,199^

Quercetin has been reported to have different mechanisms of action on ROS.

- It exhibits strong antiradical properties against radicals such as hydroxyl radical, peroxide and superoxide anion.
- It reduces superoxide anion production with xanthine oxidase.
- Cyclooxygenase and lipoxygenase inhibit enzyme activities.
- It forms a chelate with metals such as iron and copper.^198^

There are quercetin applications for Cd toxicity to remove oxidative stress and degenerative disorders that occur in various tissues and organs of the body. Quercetin has been shown to prevent renal tubular damage and oxidative stress in rats exposed to chronic Cd. In another study, it was observed that Quercetin alleviated liver damage and oxidative stress in Cd-induced liver damage. Unsal et al suggested that Quercetin exhibited a significant protective action against Cd-induced neurotoxicity in rats via inhibiting lipid peroxidation.^199-204^

Studies have shown that antioxidants reduce Cd toxicity (Table 1).

**Table 1:** Researches in the literature to remove Cd from the body and to reduce oxidative stress

**Models**	**Study Design**	**Materials**	**Effect**	**Mechanisms and Conclusion**	**Ref.**
CdCl_2_ (25 mg/kg)	Sprague-Dawley rats	Curcumin (50 mg/kg, oral)	BUN↓, Creatinine↓, AST↓, ALT↓, Glucose↓	Curcumin has protective effect against nephrotoxicity due to Cd.	^205^
CdCl_2_ (5 mg/kg b.w)	Wistar albino rats	Vitamin E (100 mg/kg b.w) and β-carotene (10 mg/kg b.w)	GSH↑, TBARS↓, AST↓, ALT↓, Glucose ↓, BUN↓, Creatinine ↓	Vitamin E, β-carotene, and/or combinations thereof can alleviate the detrimental effects of CdCl_2_.	^206^
CdCl_2_ (5 mg/kg/d b.w)	Wistar rats	Grape seed extract (400 mg/kg/d b.w)	GSH-Px↑ CAT↑, GSH -R↑, MDA ↓	Grape seed extract has beneficial protective effects against the harmful effects of CdCl_2_ on testis.	^207^
CdCl_2_ (4.4 mg/kg/d b.w)	Wistar albino rats	Grape seed extract (100 mg/kg/d b.w)	GSH-Px↑ CAT↑, GSH↑, SOD↑, MDA↓, TAC↑	Grape seed extract has protective effects on the spleen of rats by alleviating oxidative stress.	^208^
CdCl_2_ (5 mg/kg b.w)	Wistar albino rats	Sinapic acid (10 or 20 mg/kg b.w)	CAT↑, MDA↓,	Cd protects against the nephrotoxicity caused by sinapic acid.	^209^
CdCl_2_ (5 mg/kg b.w)	Sprague-Dawley rats	Thymoquinone (40 mg/kg/d)	GSH↑, SOD↑, MDA↓,	Thymoquinone prevents the harmful effects of Cd exposure on the kidneys.	^210^
CdCl_2_ (7 mg/kg b.w)	Male mice	Curcumin (50 mg/kg b.w) Resveratrol (20 mg/kg b.w) Melatonin (12 mg/kg b.w)	CAT↑, GSH↑, MDA↓ GSH-Px↑	Cd-induced toxicity has been shown to protect against lipid peroxidation of curcumin, resveratrol and melatonin treatment and to reduce the negative effect of Cd on antioxidant enzymes.	^211^
CdCl_2_ (2.5 mg/kg b.w.)	Wistar strain albino rats	Hydroxytyrosol (2-(3,4-dihydroxyphenyl) ethanol, DPE) (9.0 mg/kg b.w.) or MnCl_2_ (2.0 mg/kg b.w.)	SOD↑, CAT↑, GSH-Px↑	Manganese and DPE provide a positive contribution to reducing the damage caused by Cd toxicity. In addition, the antioxidant properties exhibited by the DPE in the liver are protective against Cd damage.	^8^
CdCl_2_ (6.5 mg/kg b.w)	Wistar albino rats	*Physalis peruviana* L. (200 mg/kg b.w)	GSH-Px↑, CAT↑, GSH↑, GR↑, MDA (TBARS)↓	*Physalis peruviana L.* is protective against acute Cd neurotoxicity.	^212^
CdCl_2_ (200 µg/mL)	Sprague-Dawley rats	1 % Taurine, 0.02 % Melatonin, 0.5 % NAC and 1 % 4 Taurine, 0.08 % Melatonin, 2 % NAC	CAT↑, SOD↑, MDA↓, GST↑, MPO↑	Taurine, melatonin and N-acetylcysteine have been shown to have some protective effect against Cd accumulation in brain and heart tissues.	^213^
CdCl_2_ (50 mg/kg b.w)	Male Wistar rats	Rutin, 25, 50 or 100 mg/kg b.w	GSH-Px↑, CAT↑, GSH↑, SOD↑, MDA↓	Rutin has protective effect against oxidative stress by stimulating antioxidant enzymes in Cd-induced testicular damage.	^214^
CdCl_2_ (20 mg/kg/b.w)	Male Wistar rats	Vit-E (20 mg/b.w) Ca + Zn (2 mg/kg)	GSH-Px↑, CAT↑, GSH↑, SOD↑, GR↑, LPO (MDA)↓	Vit-E supplementation against Cd-induced nephrotoxicity can be an important safeguard.	^215^
CdCl_2_ 2 mg/kg/d b.w)	Albino rats	Sodium selenite (1 mg/kg b.w)	MDA↓, GST↑, GSH-Px↑	Selenium treatment protects kidney tissues against Cd toxicity.	^216^
CdCl_2_ (1 mg/kg b.w)	Wistar rats	Royal Jelly (100 mg/kg b.w/day)	SOD↑, GR↑, MDA↓	Royal Jelly Cd-induced male infertility can also be a natural preservative.	^217^
CdCl_2_ (2 mg/kg/day)	Male C57 BL/6J mice	Flavocoxid (20 mg/kg/d)	GR↑, GSH↑, GSH-Px↑, PC↓	Flavocoxid significantly reduces Cd-induced oxidative damage in the kidney.	^218^
CdCl_2_(2, 4, and 8 mg/kg b.w)	Male mice	Quercetin (75 mg/kg)	GSH-Px↑, GSH↑, SOD↑, MDA↓	Quercetin has a protective effect against Cd by reducing lipid peroxidation with its anti-oxidative property.	^219^
CdCl_2_ (1.8 mg/kg)	Mice	Extra virgin olive oil (2 mL/kg b.w)	GSH-Px↑ CAT↑, SOD↑, MDA↓,	Extra virgin olive oil has been shown to be an important agent in reducing oxidative stress in the prevention of Cd toxicity.	^220^
CdCl_2_ (5 mg/kg)	Male albino rats	*Origan ummajorana* L. 1000 mg/kg b.w.	CAT↑, GSH↑, SOD↑, TBARS (MDA)↓,	Origanum majorana L. has protective properties against Cd-induced hepatotoxicity and nephrotoxicity.	^221^
CdCl_2_ (6.5 mg/kg)	Male Wistar rats	*Fragaria ananassa Crude Extract* (*250 mg/kgb.w)*	GSH-Px↑, CAT↑, GSH↑, SOD↑, MDA (LPO)↓	The crude extract of *Fragaria ananassa* has been shown to be protective against Cd-induced oxidative stress.	^147^

CdCl2: Cadmium chloride, MDA: Malondialdehyde, MPO: Myeloperoxidase, TAC: Total antioxidant capacity, LPO: Lipid peroxidation, TBARS: Thiobarbituric acid reactive substances, SOD: Superoxide dismutase, CAT: Catalase, GSH-Px: Glutathione peroxidase GR: Glutathione reductase, GSH: Glutathione, PC: Protein carbonyl.

## Conclusion and Future Perspective

Cd exposure is present in many stages of human life and has become a global problem. Cd affects skeletal, urinal, reproductive, cardiovascular, central and peripheral nerves and respiratory system in human body. If it is almost impossible to escape from Cd in the industrialized world, then we must find ways to remove heavy metals like Cd from the body. The most commonly used agent in Cd poisoning is EDTA. In addition, chelators such as penicillamine, dimercaprol (British anti-Lewisite, BAL) and dithiocarbamates are used. BAL is more toxic than its derivatives, DMPS and DMSA, and is rarely used clinically.^222,223^ The antioxidant balance of the body is greatly influenced by the diet. Pathological conditions can occur when the defenses of the body are destroyed due to nutritional deficiencies. Cd increases the ROS level and causes oxidative stress.The increase in ROS and inadequacy of defense systems cause the deterioration of antioxidant balance in the body and the formation of “oxidative stress” conditions. Antioxidants interact with free radicals, rendering them ineffective and preventing damage. They are also known as free radical scavengers. To neutralize free radicals, the body produces some endogenous antioxidants. However, there are also exogenous antioxidants taken with nutrition. Fruits, vegetables and some medical plants are rich sources of antioxidants. It has been reported that the toxic effects of Cd can be protected through antioxidants. Many antioxidant substances have been tested to minimize the oxidative stress that occurs in tissues due to Cd. The reason researchers conduct antioxidant trials is that they appear purer and more reliable. As a result of our research, we found that some antioxidants decreased Cd-induced oxidative stress. However, to elucidate future therapeutic advances, new efforts are needed to remove Cd and reduce oxidative damage, particularly to obtain appropriate doses and more understandable results.

## Ethical Issues

Not applicable.

## Conflicts of Interest

The authors declare no conflict of interest.
